# An Investigation on the Tribological Performances of the SiO_2_/MoS_2_ Hybrid Nanofluids for Magnesium Alloy-Steel Contacts

**DOI:** 10.1186/s11671-016-1546-y

**Published:** 2016-07-15

**Authors:** Hongmei Xie, Bin Jiang, Bo Liu, Qinghang Wang, Junyao Xu, Fusheng Pan

**Affiliations:** State Key Laboratory of Mechanical Transmissions, College of Materials Science and Engineering, Chongqing University, Chongqing, 400044 China; College of Mechanical and Electrical Engineering, Yangtze Normal University, Chongqing, 408100 China; Chongqing Academy of Science and Technology, Chongqing, 401123 China; Chongqing Chang-an Automobile Co., Ltd, Chongqing, 400023 China

**Keywords:** SiO_2_/MoS_2_ hybrid nanoparticles, Lubricant additive, Magnesium alloy, Friction and wear

## Abstract

Hybrid nano-materials offer potential scope for an increasing numerous novel applications when engineered to deliver availably functional properties. In the present study, the SiO_2_/MoS_2_ hybrid nanoparticles with different mass ratios were employed as lubricant additives in the base oil, and their tribological properties were evaluated using a reciprocating ball-on-plate tribometer for magnesium alloy-steel contacts. The results demonstrate that the SiO_2_/MoS_2_ hybrid nanoparticles exhibit superior lubrication performances than individual nano-SiO_2_ or nano-MoS_2_ even in high load and diverse velocity cases. The optimal SiO_2_/MoS_2_ mixing ratio and the concentration of SiO_2_/MoS_2_ hybrid nanoparticles in the base oil are 0.25:0.75 and 1.00–1.25 wt%, respectively. The excellent lubrication properties of the SiO_2_/MoS_2_ hybrid nanoparticles are attributed to the physical synergistic lubricating actions of nano-SiO_2_ and nano-MoS_2_ during the rubbing process.

## Background

Magnesium and its alloys are promising materials in transportation, electronics industries, or aerospace for their excellent properties, such as low density, high specific strength, and electromagnetic compatibility [1, 2]. Nowadays, magnesium alloy products are mainly fabricated by casting and die-casting [3]. However, the poor mechanical properties of the cast alloys are not sufficient to meet the demand of most load-bearing structural components. Compared with casting products, wrought magnesium alloys fabricated by plastic deformation processes, such as rolling, extrusion, and forging, seem to be more attractive owing to their competitive productivity and performance [4]. It should be noted that the tribological interaction always takes place during forming process as two contact surfaces (tool steel and the metal) move relative to each other. These create a great challenge to achieve high-quality product and extended tool life [5].

The use of liquid lubricants to decrease friction is an efficient way to improve energy efficiency and mechanical durability, especially in the case of magnesium and its alloys. Unfortunately, so far, there are no suitable forming fluids for the forming process of Mg alloy, even at some conditions, the forming fluid used for Al alloy forming is casually used and the result is not satisfied. The application of conventional oil-based lubricant in Al alloy forming processes relies heavily on sulfur-, chlorine-, and phosphorous-containing additives. These additives form easily sheared tribo-layers on metal surfaces, thus controlling friction and reducing wear [6]. However, the fast chemical degradation of these additives during application is accompanied by a loss of their lubrication performances. Even worse, the abovementioned additives cause negative effects on the environment even at low concentrations during the disposal of waste fluids [7]. Therefore, the exploration of the harmless lubricant with the excellent tribological performances still continues. Some efforts have already been made in order to find novel lubricant additives for magnesium alloy, such as N-containing compounds [8], borates [9], and ionic liquids [10]. All of them as lubricant additives are capable of forming tribo-layers with low shear strength to protect the contacting surfaces from damage, thereby improving the lubrication performances. Nonetheless, some problems are associated with their applications. For instance, many amide-based friction reducing agents for lubricating oils, including straight-chain amides, are not liquid at low temperature [11]. The borate without active elements, i.e., nitrogen, sulfur and chlorine, cannot be used as good lubricant additive for magnesium alloy. Additionally, boron is an electron-deficient element and has a great affinity with oxygen borate esters susceptible to hydrolysis in the presence of moisture [12]. The high cost and tedious procedure for the preparation of ionic liquids are main problems to put them into industrial application after 10 years of extensive development [13]. These disadvantages are key problems to replace the traditional phosphors- and chlorine-containing additives with the aforementioned additives.

Over the past few years, the addition of nanoparticles as lubricant additives into base fluids is a rapidly progressing field of research because nanoparticles are different from traditional bulk materials due to their extremely small size, high specific surface area, and variety of particle chemistries [14, 15]. Meanwhile, due to the tiny usage of nanoparticle additive, the negative influence on the environment is greatly suppressed [16–18]. Among of the nanoparticles, the layered nanomaterials with different shapes and morphologies, such as MoS_2_ nanoparticles, are widely used as additives to liquid lubricants [19]. Previous studies have shown that MoS_2_ nanoparticles can effectively reduce the friction in the boundary lubrication for steel/steel contacts and titanium alloy/steel pairs based on the formation of diverse types of tribo-film [20, 21]. Moreover, it was also reported that MoS_2_ nanoparticles can decrease the friction and wear even on relatively inert surfaces, such as diamond-like carbon (DLC) film [22, 23]. Despite many attempts, the combination of nano-MoS_2_ with other nanoparticles or compounds is of particular significance in terms of their application as lubricant additives because the combinations usually exhibit more prominent lubrication performances in contrast with individual nanoparticles attributed to the synergistic effect among two or more components. In this respect, Kunhong Hu et al. [24] synthesized TiO_2_/MoS_2_ nano-clusters and the tribological properties of the as-prepared TiO_2_/MoS_2_ nano-clusters as lubricant additive were investigated using a four-ball tribometer. It was found that the MoS_2_/TiO_2_ (2:1) nano-clusters achieve the lowest friction coefficient (*μ* = 0.045), which is 30.8 and 40 % lower than pure MoS_2_ and pure TiO_2_, respectively. Yanbin Zhang et al. [25] reported the use of carbon nanotubes (CNT)/MoS_2_ hybrid nanoparticles in minimum quantity lubrication for Ni-based alloy grinding. The results demonstrated that lower grinding forces and better ground surface were achieved by CNT/MoS_2_ hybrid nanofluids. Moreover, the optimal MoS_2_/CNT mixing ratio and nanofluid concentration are 2:1 and 6 wt%, respectively. Yufu Xu et al. [26] investigated the tribological behaviors of esterified bio-oil (EBO) and EBO containing graphene or/and MoS_2_ for steel/steel pairs by a point contact undirectional sliding tribometer. A synergistic effect on friction reduction and wear protection was observed with the graphene/MoS_2_ hybrids when added into EBO. These researches primarily demonstrate that MoS_2_-based hybrid nanoparticles have great potential as lubricant additives and are worth carrying out further study. As compared with the aforementioned nanoparticles, the SiO_2_ nanoparticles have attracted a great deal of research attention as a lubricant additive owing to excellent tribological performances, low cost, and facile preparation. Previous investigations about the lubrication properties of SiO_2_ nanoparticles mainly focused on Al alloy forming process, such as Al alloy machining and drilling [27, 28]. Experimental results showed that SiO_2_ nanofluids cause a decrease in friction coefficient and increase in surface quality of workpiece during machining process. This effect has been attributed to the rolling action of nanoparticles between the contact surfaces. In our early work, the tribological behaviors of nano-SiO_2_ and nano-MoS_2_ as lubricant additives for magnesium alloy/steel pairs have been studied and found that nano-MoS_2_ achieves better anti-wear behavior than nano-SiO_2_, while nano-SiO_2_ possesses better friction reducing behavior than nano-MoS_2_ [29]. In view of their respective special tribological characteristics, it is worth to study how the two particles behave together as lubricant additive and try to further optimize the comprehensive tribological performances.

The aim of the current study is to provide an effective SiO_2_/MoS_2_ hybrid nanoparticles additive for the development of magnesium alloys forming fluid. The tribological behaviors of the SiO_2_/MoS_2_ hybrid nanoparticles were investigated by considering the friction coefficient and wear volume. Further, the lubrication mechanism was discussed in detail.

## Methods

### Materials

**Fig. 1:**
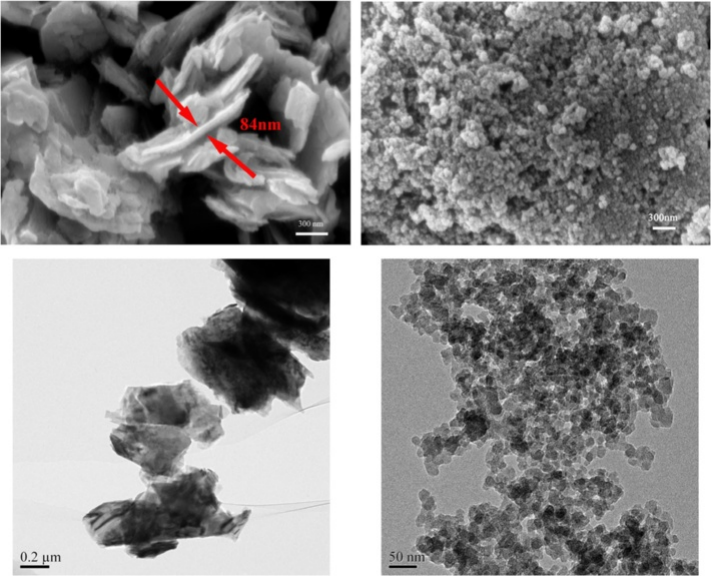
FESEM and TEM images of nano-MoS_2_ (*left*) and nano-SiO_2_ (*right*)

EOT5# engine oil (5.11 mm^2^/s at 40 °C and 0.856 g/cm^3^ at 15 °C) was adopted as base oil in the tribological investigation because it is typical oil for nonferrous metal cold forming applications. According to the supplier (Hasitai Lubricant Co., Ltd Shanghai, China), the EOT5# lubricant oil is free from sulfur, phosphorus, chlorine, and other additives. The authors deliberately chose to use a lubrication of free additives as base oil, so as to isolate the effects of the nanoparticles additives. Moreover, the use of sulfur-, chlorine-, and phosphorus-containing compounds should be restricted for environmental reasons. The nano-MoS_2_ and nano-SiO_2_ used in this study were procured commercially from Nanjing Emperor Nano Material Co. Ltd, Nanjing, China. The morphology of the two samples was investigated with Zeiss AURIGA field emission scanning electron microscope (FESEM) and JEM 1200EX transmission electron microscopy (TEM) as shown in Fig. 1. It can be observed that the nano-MoS_2_ possesses a flake-like shape mainly with above 300 nm in length and about 90 nm in thickness, and the nano-SiO_2_ keeps a spherical micro-structure with a mean diameter of 30 nm. The nanofluids were prepared as follows: nano-SiO_2_, nano-MoS_2_ and their hybrids, with different concentrations (mass fraction) of nano-SiO_2_ and nano-MoS_2_, were separately dispersed into the base oil through ultrasonication for 2 h to obtain a series of homogeneous suspensions.

### Friction and Wear Test

The friction reduction and anti-wear abilities of nanofluids were evaluated using a reciprocating ball-on-plate friction and wear tester (CSM Instruments, Peseux, Switzerland). Throughout the test, the upper AISI 52100 steel ball with diameter of 6 mm and surface roughness (Ra) of 0.05 μm slides against the lower fixed AZ31 magnesium alloy plates. The starting billet of commercial as-cast AZ31 magnesium alloy (Mg-3.07Al-0.78Zn-0.38Mn in wt%) with 82 mm in diameter was homogenized at 380 °C for 2 h. Subsequently, this ingot was extruded to the sheet of 3-mm thickness (normal direction, ND) and 56-mm width (transverse direction (TD)) at 380 °C. Meanwhile, the speed of extrusion process was 20 mm/s with the extrusion ratio of 34:1. The mechanical properties of the extruded AZ31 Mg alloy are provided in Table 1.

**Table 1:** Mechanical properties of extruded AZ31 magnesium alloy used in this study

Material	0.2 % YS/MPa	UTS/MPa	Elongation/%	HV_0.01_
Extruded AZ31	142.1	305	18.5	66.7

The as-extruded sheet was cut into samples with dimensions of 10 mm (TD) × 20 mm (ED) × 3 mm (ND). Prior to examination, the samples were polished with 1000 grit silicon carbide paper to a mean surface roughness of Ra ~0.08 μm. The ball slides at a stroke of 6 mm back and forth along extrusion direction. In the first group of experiments, the influence of the SiO_2_/MoS_2_ mixing ratio on the lubrication behaviors of nanofluids was discussed. The normal load during the tribological tests was 8 N corresponding to maximum Hertzian contact stress 446 MPa of at least 50 % higher than the yield strength of magnesium alloy sheets. The sliding speed was 30 mm/s, and the sliding test duration was 1.5 h. Lubricant oil was supplied to the top of the plate before testing, covering the entire surface. Friction coefficient and wear volume were used as characterization parameters. Five different SiO_2_/MoS_2_ mixing ratios were designed: pure MoS_2_, SiO_2_/MoS_2_ (0.25:0.75), SiO_2_/MoS_2_ (0.5:0.5), SiO_2_/MoS_2_ (0.75:0.25), and pure SiO_2_. The concentration of these additives in the base oil was 1 wt%. The optimal SiO_2_/MoS_2_ mixing ratio was obtained from the first group of experiments. However, the concentration of nanoparticles in the base fluids is another fundamental issue because excessive concentration will make nanoparticles agglomeration and thereby destroy lubrication properties. In the second group of experiments, the influence of the concentration of SiO_2_/MoS_2_ hybrid nanoparticles on lubrication properties was discussed by changing the mass fraction of the SiO_2_/MoS_2_ hybrid nanoparticles in the base oil. The nanofluids were prepared as 0.25, 0.50, 0.75, 1.00, 1.25, 1.50, and 2.00 % by weight of SiO_2_/MoS_2_ hybrid nanoparticles into the base oil. The optimal concentration was determined. In the third group of experiments, the influence of normal load and sliding speed on the lubrication properties of the fluids was performed with a normal load varying from 1 to 10 N (corresponding maximum Hertzian contact stress from 223 to 480 MPa) and a sliding velocity ranging from 10 to 80 mm/s for 30 min. The friction coefficient was recorded by the tribometer in real time. Three sets of tests at the same normal pressure and sliding velocity were conducted to obtain each datum point to verify the repeatability and accuracy of the test results. Using nanofluids, the wear of the AISI 52100 steel ball was not measurable and there was almost no variation in the roughness measurements. For this reason, the wear volume of AZ31 magnesium alloy plate was calculated to evaluate the lubricant effectiveness in wear reduction in the overall lubricating system.

### Characterization

The morphologies of the worn surfaces were determined by Zeiss AURIGA FESEM. The wear depth and wear track profiles after the friction tests were obtained by a noncontact 3D surface profiler (Olympus OLS4000), and the wear volume of the plates was calculated from the wear depth. The final values quoted for the wear volume of the specimen were averages of three tests results. The chemical compositions of the worn surfaces were characterized by a VG model Escalab 250 X-ray photoelectron spectroscopy (XPS) with Al-Kα radiation as the excitation source. And the binding energy of C1s at 284.6 eV was utilized as the reference. Prior to the analysis, the specimens were cleaned ultrasonically for 5 min with acetone, in order to eliminate the residual lubricant.

## Results and Discussion

### Effect of SiO_2_/MoS_2_ Mixing Ratio on Lubrication Performance

**Fig. 2:**
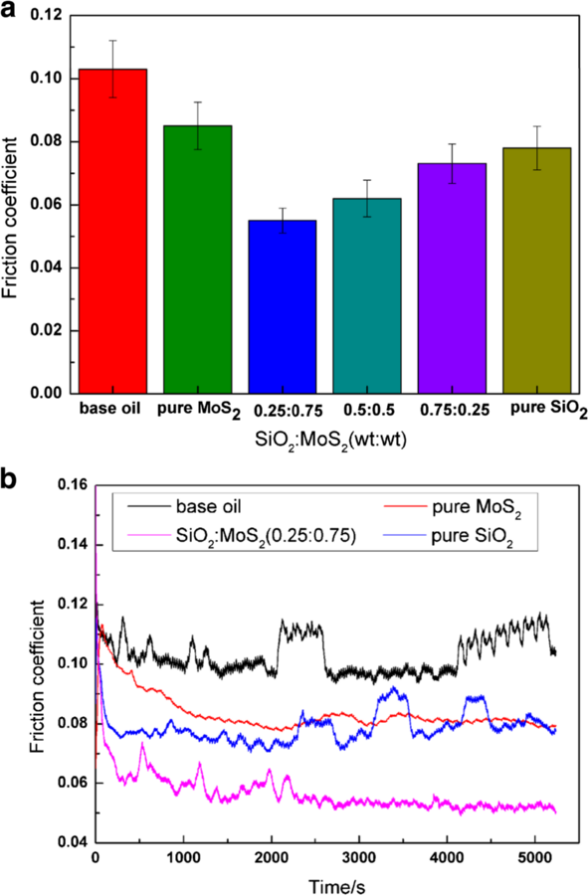
**a** Changes in the average friction coefficient as a function of different SiO_2_/MoS_2_ mixing ratios. **b** Evolution of the friction coefficient with the sliding time (8 N, 30 mm/s, 1.5 h)

**Fig. 3:**
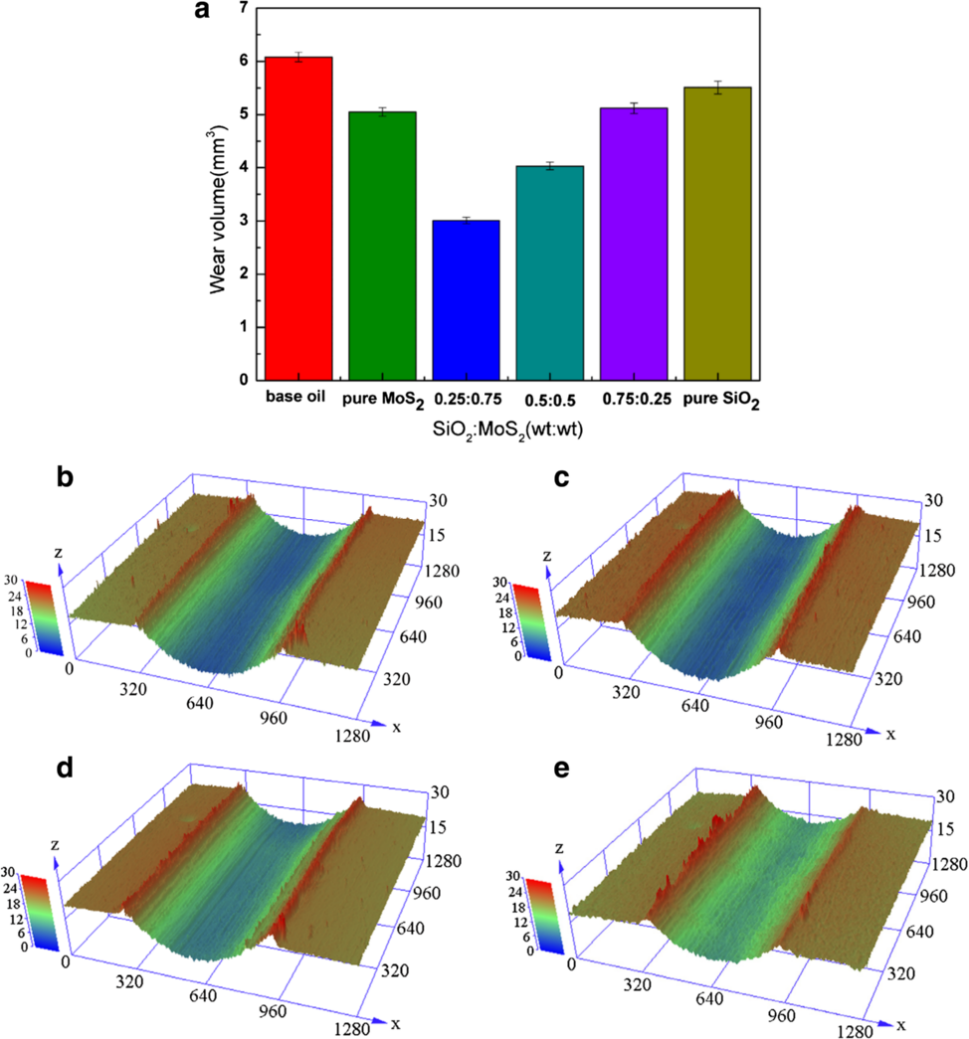
**a** Changes in the average wear volume as a function of different SiO_2_/MoS_2_ mixing ratios and 3D optical microscopic images of the wear tracks corresponding to **b** base oil, **c** pure SiO_2_, **d** pure MoS_2_, and **e** SiO_2_/MoS_2_ (0.25:0.75) (8 N, 30 mm/s, 1.5 h)

Figures 2 and 3 display the friction and wear results of the SiO_2_/MoS_2_ hybrid nanofluids for magnesium alloy/steel contacts at 8 N and 30 mm/s for 1.5 h, compared with those of pure nanofluids and neat oil without nanoparticles tested in the same conditions. Among the six prepared fluids, the base oil gives the highest friction coefficient (*μ* = 0.103). The changes in sliding time have significant effect on the friction coefficient of the base oil. It can be seen that the friction coefficient of the base oil shows considerable oscillation during the test. The friction coefficient of pure MoS_2_ nanofluids and pure SiO_2_ nanofluids are 0.085 and 0.078, respectively. It is evident from these measurements that pure nanoparticles as lubricant additive have positive functions for improving the friction reduction performance of the base oil. As the increase of test duration, the friction coefficient of pure SiO_2_ nanofluids varies in the range of 0.07–0.09, accompanied by severe oscillation. While for the pure MoS_2_ nanofluids, the friction coefficient decreases from an initial value of ∼0.11 to ∼0.08 after rubbing for 1000 seconds and remained essentially constant thereafter. This result proves that the pure MoS_2_ nanofluids have much better friction reduction properties than pure SiO_2_ nanofluids at a longer test time. However, the reduction of friction coefficient is more significant for the three SiO_2_/MoS_2_ hybrid nanofluids when compared to that found with the pure nanofluids. The improvement of lubrication performances should be ascribed to the synergistic lubricating effect of the hybrid nanoparticles during sliding. The friction coefficient of the SiO_2_/MoS_2_ hybrid nanofluids decreases with increasing mixing ratio of SiO_2_ to MoS_2_ until a mass ratio of 0.25:0.75 (SiO_2_/MoS_2_) is achieved. With the further increase of mixing ratio of SiO_2_ to MoS_2_, the friction coefficient gradually increases. The SiO_2_/MoS_2_ (0.25:0.75) hybrids achieve the lowest friction coefficient (*μ* = 0.055), which is 35.3 and 29.5 % lower compared with pure MoS_2_ and pure SiO_2_, respectively. In addition, the friction coefficient of the SiO_2_/MoS_2_ (0.25:0.75) hybrid nanofluids shows a slightly fluctuation at the initial stage of the test and then decreases and stabilizes gradually in the latter part of the curve. The excellent lubricating performances of SiO_2_/MoS_2_ (0.25:0.75) hybrid nanofluids indicate that the synergistic lubricating effect of nano-SiO_2_ and nano-MoS_2_ are closely related to the mixing ratio of SiO_2_ to MoS_2_ in the base oil.

Figure 3 shows average wear volume and 3D optical microscopic images of the wear tracks lubricated with different samples. The variation of wear volume is similar to the change in friction coefficient in Fig. 2. In this respect, the SiO_2_/MoS_2_ (0.25:0.75) hybrid nanofluids also outperform the rest samples. In comparison with the base oil, the SiO_2_/MoS_2_ (0.25:0.75) hybrid nanofluids lead to a significant reduction of wear volume by 50.5 % (from 6.08 to 3.01 mm^3^ in mean value). In contrast, the pure MoS_2_ nanofluids and SiO_2_ nanofluids are less effective by offering 17 and 9.4 % wear volume reduction in contrast with the base oil (from 6.08 to 5.05 and 5.51 mm^3^ in mean value), respectively. Comparative results of friction coefficient and wear volume under different SiO_2_/MoS_2_ mass ratio further prove the lubrication advantages of the SiO_2_/MoS_2_ (0.25:0.75) hybrid nanofluids.

### Effect of the Concentration of Hybrid Nanoparticles on the Lubrication performance

**Fig. 4:**
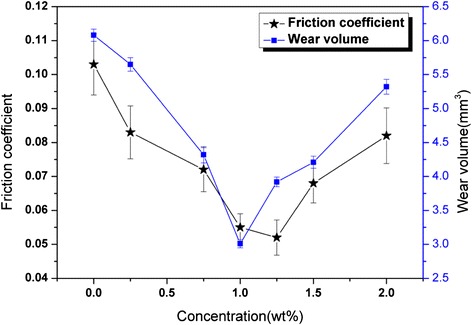
Variations of the friction coefficients and wear volume of the plates lubricated with the SiO_2_/MoS_2_ (0.25:0.75) hybrid nanofluids as a function of SiO_2_/MoS_2_ (0.25:0.75) hybrid nanoparticles concentration (8 N, 30 mm/s, 1.5 h)

It can be concluded from the previous experiments that 0.25:0.75 is the optimal SiO_2_/MoS_2_ mixing ratio of hybrid nanoparticles. In this group of experiments, the effect of the SiO_2_/MoS_2_ (0.25:0.75) hybrid nanoparticles concentration on the lubrication properties was investigated by changing the mass fraction (from 0.25 to 2 wt%) of the SiO_2_/MoS_2_ hybrid nanoparticles in the base oil. Figure 4 shows variations in the friction coefficient and wear volume with concentration of the SiO_2_/MoS_2_ (0.25:0.75) hybrid nanoparticles in the base oil at 8 N and 30 mm/s for 1.5 h. It can be observed that both friction coefficient and wear volume illustrate a nearly similar pattern with increasing concentration of the SiO_2_/MoS_2_ hybrid nanoparticles in the base oil. Both friction coefficient and wear volume decrease initially and then increase with increasing concentration of the SiO_2_/MoS_2_ hybrid nanoparticles. The lowest friction coefficient emerges at the concentration of 1.25 wt%, and the smallest wear volume appears at the concentration of 1.00 wt%. Therefore, the optimal concentration of SiO_2_/MoS_2_ hybrid nanoparticles in the base oil is 1.00–1.25 wt%. Nanoparticle agglomeration occurs once this optimum concentration is exceeded, which will influence the lubrication efficacy [30]. On the one hand, nanoparticle agglomeration destroys the integrity of the lubrication film. On the other hand, it is also considered that the increased number of nanoparticles clusters sliding against each other can have increasing influence on friction coefficient and wear volume [31]. Based on the observations on the tribological behaviors of the SiO_2_/MoS_2_ hybrid nanofluids and cost savings, 1.00 wt%, concentration of the SiO_2_/MoS_2_ hybrid nanoparticles in the base oil was chosen for further testing.

### Effect of Normal Load and Sliding Speed on Lubrication Performance

**Fig. 5:**
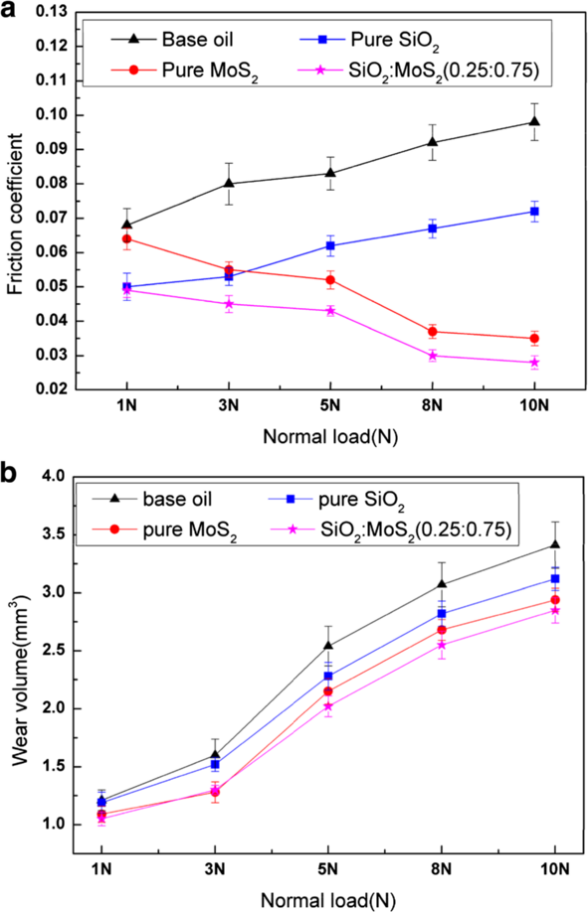
Effects of loads on **a** average friction coefficient and **b** wear volume of magnesium alloy specimens lubricated by the base oil with and without additives (sliding speed 80 mm/s; testing time 0.5 h)

The effect of the normal load (from 1 to 10 N) on the lubrication properties of the 1.00 wt% SiO_2_/MoS_2_ (0.25:0.75) hybrid nanofluids was studied for magnesium alloy/steel contacts at 80 mm/s for 30 min, compared with those of pure nanofluids and neat oil without nanoparticles tested in the same conditions. The mean friction coefficient and wear volume are reported in Fig. 5. It can be observed that the average friction coefficient for base oil and pure SiO_2_ nanofluids increases gradually with the increase of load. Higher loads can lead to smaller micro-intervals between the friction pairs. There is less base oil at a molecular level that could be drawn into the frictional interface, thus increasing friction coefficient [32]. In case of pure SiO_2_ nanofluids, spherical SiO_2_ nanoparticles are more likely to roll between the rubbing surfaces, thus reducing the friction coefficient [33]. However, it is necessary to emphasize that the rolling effect of spherical nanoparticles during rubbing is closely related to the thickness of the lubricant film. It has been reported in the literature [34] that when the thickness of the lubricant film is close to the size of the spherical nanoparticles, the shape of the nanoparticles is preserved. In our study, the corresponding minimum film thickness between two surfaces can be approximately predicted by the Hamrock-Dowson equation [35]. The thickness of lubricant film at the point of contact is 44, 35, 31, 28, and 26 nm for 1, 3, 5, 8, and 10 N, respectively. This would indicate that the thickness of the lubricant film between the friction pairs decreases with the increase of the load and is getting close to the size of the spherical SiO_2_ nanoparticles. The shape of the nanoparticles is preserved during sliding, thereby increasing friction coefficient. The friction behaviors of the pure MoS_2_ nanofluids and the SiO_2_/MoS_2_ hybrid nanofluids are found to be quite different from the base oil and pure SiO_2_ nanofluids under the same test conditions. After the nano-MoS_2_ or SiO_2_/MoS_2_ hybrid nanoparticles are added into the base oil, the average friction coefficient decreases gradually with load increasing. During sliding process, part of nano-MoS_2_ diffused into the friction region and took tribo-chemistry reaction with rubbing surface to generate a tribo-chemical reaction films which can protect magnesium alloy surface effectively. The forming velocity of tribo-chemical reaction film is closely related to the energy supplied during the rubbing process. With the load increasing, more energy will be provided and the nano-MoS_2_ becomes easier to react with the new-exposed friction pair surfaces. This contributes to the formation of the tribo-chemical reaction film on the surface, and thus resulting in friction reduction [36]. However, the wear volume of all samples maintains a similar trend, increasing gradually with the increase in normal load. At lower loads, the anti-wear behavior is marginally different with all the nanofluids reporting similar wear volume. However, with the increased load the difference in the anti-wear behavior is substantial. Moreover, the wear volume of the SiO_2_/MoS_2_ hybrid nanofluids is significantly lower than that of the rest samples at high load. The different friction coefficient and wear volume can be attributed to the formation of disparate adsorption film and/or tribo-chemical reaction film on the rubbing metal surface. Detailed information on the composition of the films formed on the rubbing surfaces is presented in the “Rubbing Surface Analyses section.”

**Fig. 6:**
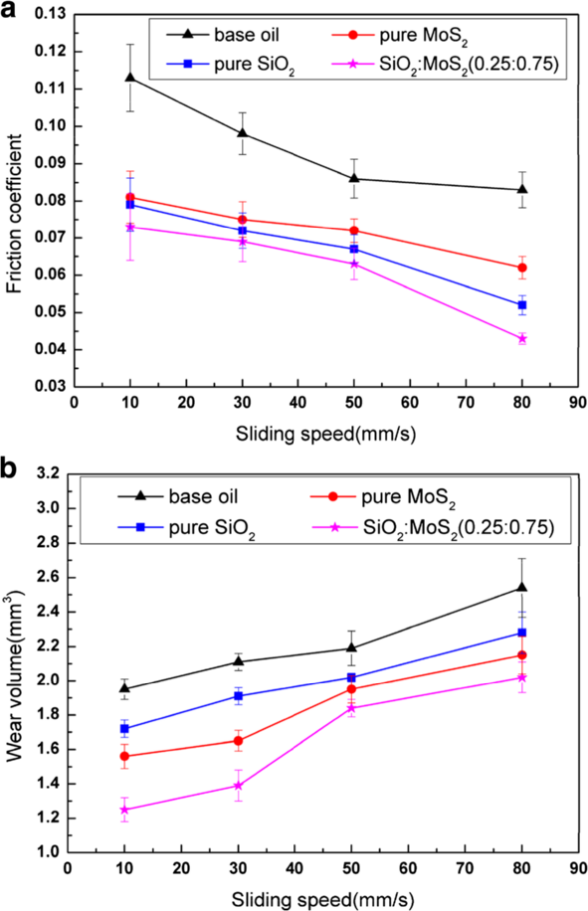
Effects of sliding speeds on **a** average friction coefficient and **b** wear volume of magnesium alloy specimens lubricated by the base oil with and without additives (normal load 5 N; testing time 0.5 h)

During the tests, the sliding speed can make another significant impact on the lubrication performances. Figure 6 describes the variations of the friction coefficients and wear volumes of the plates lubricated with the SiO_2_/MoS_2_ hybrid nanofluids as a function of the sliding speed, compared with those of pure nanofluids and neat oil without nanoparticles tested in the same conditions. It can be observed that the friction coefficients of all the fluids gradually decreased with an increase in the sliding velocity over a wide range of 10 to 80 mm/s at 5 N for 30 min. With an increase in the sliding speed, a thicker liquid layer would be formed between the shearing surfaces, thus reducing the actual contact area and the friction force. From Fig. 6b, it can be seen that the wear of magnesium alloy specimens increased with the increase in sliding speed when lubricated by all the fluids. This can be explained by the fact that there is a longer sliding distance within the same test duration at higher sliding speeds. The most significant improvement of lubricity is obtained by the SiO_2_/MoS_2_ hybrid nanofluids for the entire range of sliding velocities. The lubricating mechanism for the SiO_2_/MoS_2_ hybrid nanofluids is further analyzed in the “Lubrication Mechanism of the SiO_2_/MoS_2_ Hybrid Nanofluids” section.

### Rubbing Surface Analyses

**Fig. 7:**
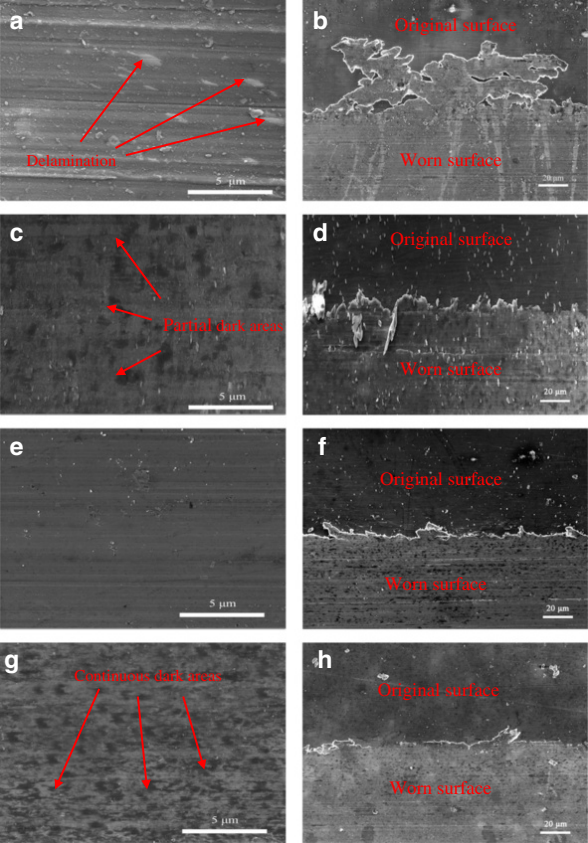
The worn surface morphologies and the edge analysis of the worn surface lubricated with **a**, **b** base oil, **c**, **d** pure MoS_2_ nanofluids, **e**, **f** pure SiO_2_ nanofluids, and **g**, **h** SiO_2_/MoS_2_ (0.25:0.75) hybrid nanofluids (8 N, 30 mm/s, 1.5 h)

Figure 7 displays the FESEM morphologies of the worn surfaces of the magnesium alloy lubricated with base oil, pure MoS_2_ nanofluids, pure SiO_2_ nanofluids, and SiO_2_/MoS_2_ (0.25:0.75) hybrid nanofluids under the load of 8 N and the speed of 30 mm/s for 1.5 h. It is clearly seen that the worn surface lubricated with the base oil (Fig. 7a) displays severe scuffing accompanied with serious delamination. Thus, severe wear occurs in this case. Moreover, the severely deformed material layers (Fig. 7b) are extruded along the sliding direction, forming flares and sizeable bulbs at the periphery of the sliding track. However, it should be mentioned that the severely deformed material layers are not observed at the periphery of the sliding track lubricated with nanofluids (Fig. 7d, f, h). Even so, the worn surfaces under the lubrication of various nanofluids are very diverse. In the presence of nano-SiO_2_ (Fig. 7c), the wear process slows down. The region of the worn surface is clean with several thin furrows, which indicates that SiO_2_ nanophases can make the lubricant system more energy efficient in contrast with the base oil. Figure 7e shows the worn surface lubricated with pure MoS_2_ nanofluids. There are a lot of small furrows and scratches on the friction surface, and a partial coverage of protection film is formed during sliding process. In contrast, the worn surface lubricated with the SiO_2_/MoS_2_ hybrid nanofluids (Fig. 7g) is quite smooth and shows only slight grooves. More importantly, the continuous dark areas can be found throughout the wear track, indicating the formation of a compact protection film. This compact protection film is believed to be responsible for the SiO_2_/MoS_2_ hybrid nanofluids’ friction reduction and anti-wear functionality. The FESEM images of worn surface were in good agreement with the measured tribological behaviors.

**Fig. 8:**
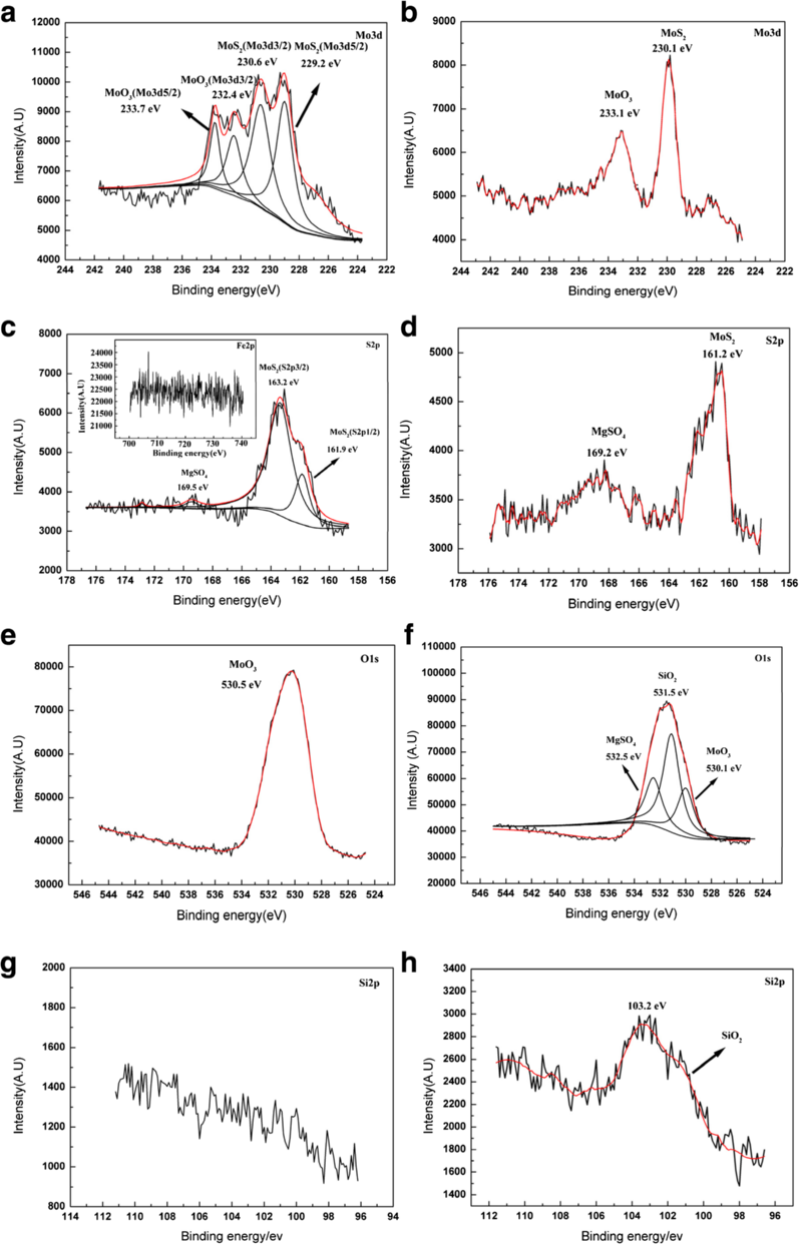
X-ray photoelectron spectra of the elements (Mo3d, S2p, O1s, Fe2p (inset of Fig. 11c), and Si2p) on the worn surface lubricated with pure MoS_2_ nanofluids (**a**, **c**, **e**, **g**) and SiO_2_/MoS_2_ (0.25:0.75) hybrid nanofluids (**b**, **d**, **f**, **h**) (8 N, 30 mm/s, 1.5 h)

The XPS analyses of Mo3d, S2p, O1s, Fe2p, and Si2p on the wear tracks lubricated with pure MoS_2_ nanofluids or SiO_2_/MoS_2_ hybrid nanofluids were further carried out to monitor the chemical changes. As shown in Fig. 8a, c, e, g concerning the rubbed surface lubricated with pure MoS_2_ nanofluids, Mo3d exists as MoS_2_ with peaks at 230.6 and 229.2 eV [37]. This may indicate that nano-MoS_2_ have physical adsorption on the worn surfaces. The additional contribution of Mo3d at higher energy, in association with the O1s peak at 530.5 eV, can be attributed to oxygenated Mo (VI) species. It is reasonable to presume that the adsorbed MoS_2_ platelets were oxidized partly into oxides of molybdenum during the rubbing process [38]. The XPS peak of the S2p spectrum appears at 163.2 eV, which corresponds to MoS_2_. Apart from MoS_2_, the weak S2p peak at higher binding energy (169.5 eV) is ascribed to MgSO_4_. Even so, the O1s signal assigned to MgSO_4_ is not observed in Fig. 8e. A possible reason for this phenomenon is that the amount of MgSO_4_ is not enough to affect the XPS spectra of O1s. Besides, the peak of S2p at 161.9 eV is difficult to be clarified because the binding energy of iron sulfide species (FeS) and molybdenum disulfide (MoS_2_) is both between 161 and 162 eV [37]. In order to determine the actual type of compound, the XPS of Fe2p was studied as shown in the inset of Fig. 8c. It can be seen that the signal of iron was not detected. Therefore, we argue that the peak of S2p at 161.9 eV comes from MoS_2_ and not FeS. For the SiO_2_/MoS_2_ hybrid nanofluids, the Mo3d spectrum appears at approximately 230.1 and 233.1 eV (Fig. 8b), which correspond to MoS_2_ (in association with S2p peak at 161.2 eV) and MoO_3_ (in combination with O1s peak at 530.4 eV), respectively. There are some differences between the samples with respect to the distribution of the oxides of molybdenum. The sample lubricated with the SiO_2_/MoS_2_ hybrid nanofluids shows less oxide present compared to the sample lubricated with pure MoS_2_ nanofluids. It suggests that the introduction of nano-SiO_2_ prevents the oxidation of the MoS_2_ on the specimen surface. The S2p spectrum for the SiO_2_/MoS_2_ hybrid nanofluids consists of two peaks (Fig. 8d). The peak at 161.2 eV is attributed to MoS_2_, and the peak located at 169.2 eV is assigned to MgSO_4_ (in association with the O1s peak at 532.5 eV). Interestingly, it can be seen from the spectra that the wear tracks lubricated with SiO_2_/MoS_2_ hybrid nanofluids show a much stronger signal for MgSO_4_ in contrast to the wear tracks lubricated with pure MoS_2_ nanofluids. The higher intensity for the SiO_2_/MoS_2_ hybrid nanofluids may lie in the fact that MoS_2_ mixed with SiO_2_ can easily react with magnesium alloy, indicating that more compact tribo-chemical film is obtained on the worn surface lubricated with the SiO_2_/MoS_2_ hybrid nanofluids. The result is also confirmed by the FESEM images in Fig. 7c. The other interesting difference is that Si2p at 103.2 eV (Fig. 8h) ascribed to SiO_2_ is found on the worn surface lubricated with the SiO_2_/MoS_2_ hybrid nanofluids [39], whereas at the same position, there are no clear signal peaks when lubricated by pure MoS_2_ nanofluids (Fig. 8g). Therefore, it can be concluded that the nano-SiO_2_ participated in the formation of the lubricating film and collaborated with the nano-MoS_2_ to lubricate the magnesium alloy surface. These results confirm that the positive effect of the SiO_2_/MoS_2_ hybrid nanofluids is more pronounced in terms of the lubrication properties when compared to that found with the pure MoS_2_ nanofluids.

### Lubrication Mechanism of the SiO_2_/MoS_2_ Hybrid Nanofluids

**Fig. 9:**
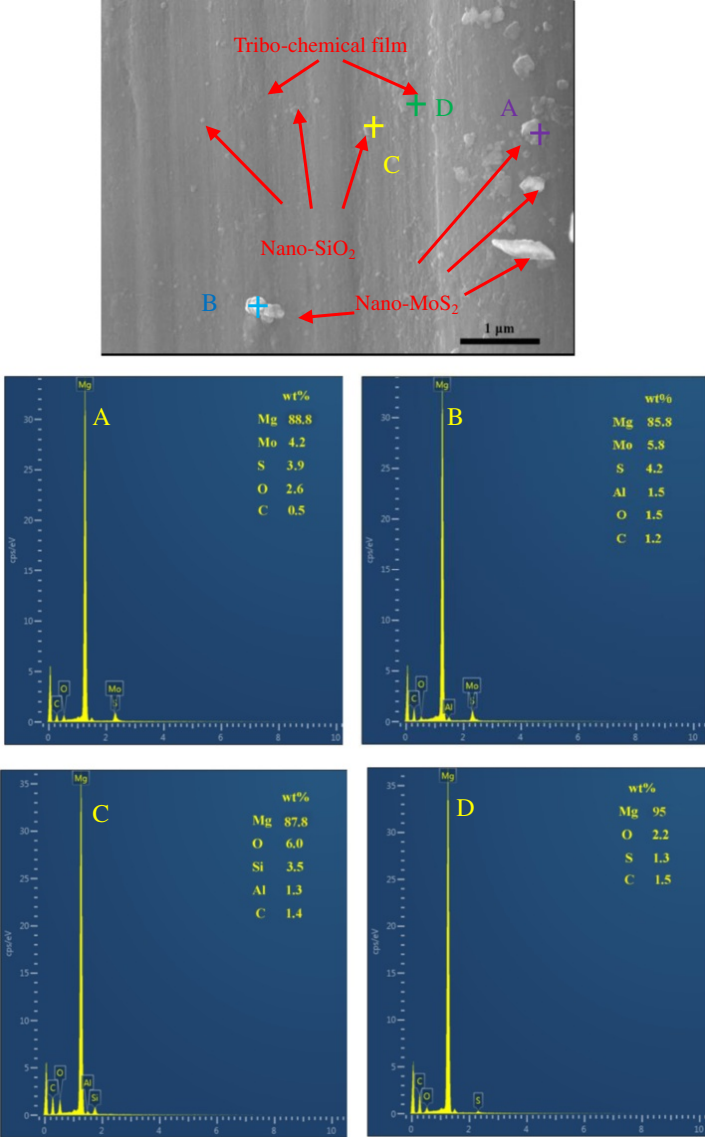
High-magnification FESEM-EDS spectra of worn plate surfaces lubricated with the 1.00 wt% SiO_2_/MoS_2_ (0.25:0.75) hybrid nanofluids (8 N, 30 mm/s, 1.5 h)

**Fig. 10:**
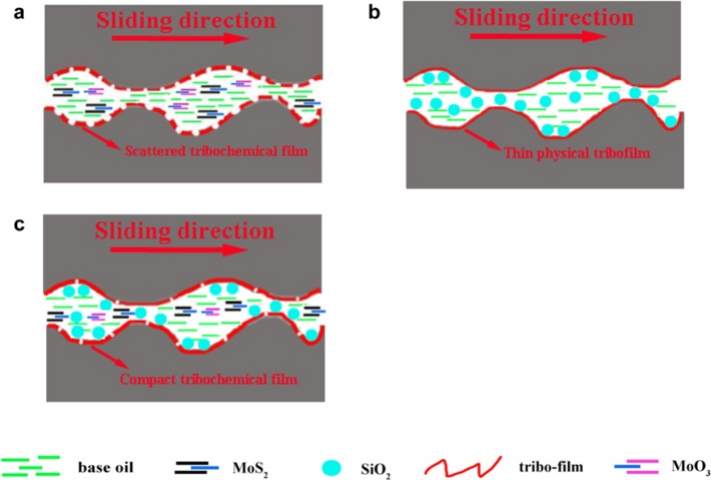
Schematic explanation of the lubricating mechanisms of **a** pure MoS_2_ nanofluids, **b** pure SiO_2_ nanofluids, and **c** SiO_2_/MoS_2_ hybrid nanofluids

**Fig. 11:**
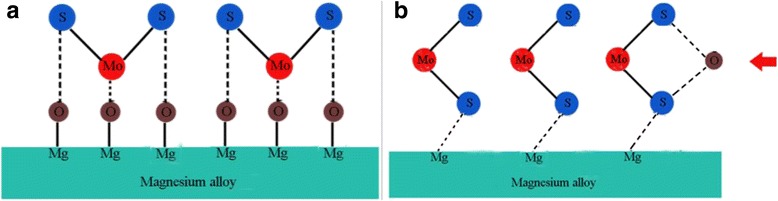
Schematic showing the possible binding mechanism of MoS_2_ on the magnesium alloy surface. **a** Bonding energy between Mg and O and between Fe and O on the basis of Pearson hardness. **b** Reaction between the nano-MoS_2_ and the Mg metal atoms

According to the friction tests run on a reciprocation ball-on-plate friction tester, it can be found that the SiO_2_/MoS_2_ hybrid nanoparticles as lubricant additive exhibit superior lubricating performances than individual nanopartcles. To understand the mechanism that is underlying the trends seen in the lubrication responses, the high magnification FESEM-EDS spectra of the worn surface lubricated with the 1.00 wt% SiO_2_/MoS_2_ (0.25:0.75) hybrid nanofluids is chosen for detailed analyses in Fig. 9. The schematic explanation of the corresponding lubricating mechanisms is summarized in Fig. 10. Figure 9 clearly shows that nano-MoS_2_ protects the surface of magnesium alloy in two ways. At first, nano-MoS_2_ is physically adsorbed on the contact surface via the flow of lubricating oil and compressive stress. Intrinsically, hexagonal lamella of nano-MoS_2_ is stacked by weak van der Waals force between layers. Low resistance to shear between the weakly interacted lamella under the sliding contact stress reduced the friction coefficient. Besides that, when a normal force exists, the MoS_2_ nanoparticle will extend into thin physical films in the contact zone. As the test time goes on, the applied load during sliding will produce high temperature and pressure. The deposited nano-MoS_2_ on the surfaces will undergo complex tribo-chemical reactions, resulting in forming a new chemical transfer film on the lubricated metal surface. Combined with the XPS spectra (Fig. 8), the tribo-chemical reaction film is composed of MoO_3_ and MgSO_4_. The tribo-chemical reaction film strongly combines with the workpiece material, which is essential for efficient long-term lubrication. The mechanism of the tribo-chemical reaction can be directly compared to those obtained in the literature concerning the interactions between MoS_2_ nanoparticles and steel surface [37]. Figure 11 is drawn to explain the possible binding mechanism of MoS_2_ on the magnesium alloy surface. Two possible mechanisms were proposed for the interactions between MoS_2_ nanoparticles and steel surface. The first is associated with a tribo-film adhesion through the bonding of molybdenum with the oxygen that was found in the native surface oxide layer of the steel. Correspondingly, a possible chemical reaction between the molybdenum and the oxygen from MgO is chemically reasonable. This is due to the fact that chemical reaction occurs between MgO and the nano-MoS_2_ could be explained by a lower bonding energy between Mg and O than between Fe and O on the basis of Pearson hardness (Fig. 11a). Another possibility is that the oxide layer would be removed during the first cycles of the friction test and the reaction between the nano-MoS_2_ and the Mg metal atoms could occur (Fig. 11b). At this stage, the precise role of the oxide layer in the mechanism of adhesion of the tribo-film on the worn surface is still extremely difficult to judge from the experiments results. Even so, it was shown in this study that a tribo-chemical reaction between the nano-MoS_2_ platelets could occur via either the native oxide layer or the metal Mg atoms during the frictional process. Besides that, further understanding the role of the protect film at disparate stage of the whole test is crucial and necessary. As shown in Fig. 2, it is apparent that the friction coefficient of pure MoS_2_ nanofluids is higher during the first 1000 s and then decreases slowly until reaches a steady state. However, this high friction coefficient period was not observable with the pure SiO_2_ nanofluids and the SiO_2_/MoS_2_ hybrid nanofluids. Similar evolution of friction coefficient with time for a steel-steel contact lubricated with pure MoS_2_ nanofluids, where a higher friction coefficient at the early stage of the test can be seen, was reported by L. Cizaire et al [40]. These authors explain that the friction coefficient is higher at the beginning of the test owing to the absent of the tribo-chemical film. However, the friction coefficient gradually decreased once the tribo-chemical film formed during the rubbing process [41]. In comparison with pure nano-MoS_2_ additive (Fig. 10a), the SiO_2_/MoS_2_ hybrid nanoparticles additive with better catalytic activity may catalyze the oxidation of S^2−^ more quickly during the sliding [42]. So the running-in time required to obtain very low friction values for SiO_2_/MoS_2_ hybrid nanoflids is shorter than pure MoS_2_ nanofluids. Even under static conditions, the chemical reaction does not stop and more and more tribo-chemical compound is generated, leading to the formation of more compact and efficient tribo-chemical film on the worn surface (Fig. 10c).

In contrast, the lubricity mechanism of spherical nano-SiO_2_ is quite different from that of nano-MoS_2_ platelets. The higher-magnification image of the worn surface in Fig. 9a and corresponding EDS reveal the presence of nano-SiO_2_ on the worn surface. Unlike nano-MoS_2_, nano-SiO_2_ possesses nano-scale size and excellent dispersion in the base lubricant. These characteristics allow the nano-SiO_2_ to easily enter the contact area, thereby resulting in faster running-in conditions (Fig. 2). The rolling effect was often proposed as a lubrication mechanism for spherical nanoparticles, although little research has been conducted on the direct visualization of the rolling behavior of the nano-SiO_2_ into the contact interfaces [33, 43]. In this way, nanoparticles decrease the shear stress and lead to the reduction in friction coefficient. Besides, even though the surface of the magnesium alloy plate was looking smooth, when we observed its micro meter and nanoscale image, the surface was complicated with ridges and valleys. When nanoparticles were added into the base oil, the filling of valleys of the contacting asperities will occur. This is also supported by the high magnification FESEM spectra (Fig. 9), which shows the distribution of nanoparticles on the worn surface. As shown in Fig. 9, it is clearly seen that nano-SiO_2_ fills up the grooves of the rubbing surfaces, while nano-MoS_2_ deposits on the flat of the worn surfaces. It demonstrates that the difference in the distribution of nanoparticles on the worn surface is substantial. This can be explained by the model proposed by Mosleh Mohsen [21]. The authors suggested that the nanoparticles can fill up valleys and smooth the surface if the characteristic length *l* of flaky nanoparticles is smaller than the peak-to-valley roughness of the harder surface which is equal to 4 Ra, i.e., the condition of *l* < 4 Ra applies. With respect to spherical nanoparticles, the diameter *d* of the particle should satisfy *d* < 0.67 Ra. Comparatively, nano-SiO_2_ with an average size of about 30 nm acts as a third body material filling in the valley of surface with 0.08-μm surface roughness, increasing real contact area. As a result, the load can be distributed over a larger contact area, so that the effective contact pressure decreases and consequently the wear also reduce. However, nano-MoS_2_ platelets may be difficult to fill in the cavities because its length (more than 300 nm) is larger than 4 Ra in our study. In comparison with pure nano-SiO_2_ additive (Fig. 10b), the micro-cooperation for SiO_2_/MoS_2_ hybrid nanoparticles additive occurred in succession between the shearing-sliding of the nano-MoS_2_ platelets structure and the filling of nano-SiO_2_ particles during sliding process, resulting in excellent lubrication performances (Fig. 10c). However, the efficiency of the SiO_2_/MoS_2_ hybrid nanofluids was influenced by the mass ratio of SiO_2_/MoS_2_. For instance, the lubrication performances of SiO_2_/MoS_2_ (0.5:0.5) and SiO_2_/MoS_2_ (0.75:0.25) were a little inferior to that of the SiO_2_/MoS_2_ (0.25:0.75) at the test condition. Namely, there was an optimum dose of nano-SiO_2_ in the SiO_2_/MoS_2_ hybrid nanofluids. If excess nano-SiO_2_ was added into the SiO_2_/MoS_2_ hybrid nanofluids, the contact area was saturated with nano-SiO_2_. Any more nano-SiO_2_ took up the space of nano-MoS_2_ and disturbed its lubrication. The nano-SiO_2_ has outstanding mechanical properties especially in terms of hardness (Vickers hardness—1000 kgf/mm^2^); therefore, the excess hard nano-SiO_2_ as an abrasive plows the soft magnesium alloy surfaces (Vickers hardness—66.7 kgf/mm^2^) under the applied load. It will facilitate the abrasion of the magnesium alloy surface during rubbing process and thus results in the worse anti-wear property. This was confirmed by the poor anti-wear behaviors of the samples with high concentrations of nano-SiO_2_, such as SiO_2_/MoS_2_ (0.5:0.5), SiO_2_/MoS_2_ (0.75:0.25) and pure nano-SiO_2_ as shown in Fig. 3a. Therefore, the nano-SiO_2_ and nano-MoS_2_ mass ratio of 0.25:0.75 is the best proportion for synergetic lubrication effect under the selected testing conditions. In addition, from the XPS results of the worn magnesium alloy surface, nano-SiO_2_ is shown to prevent nano-MoS_2_ from oxidation, resulting in increased oxidation resistance of nano-MoS_2_ during rubbing process. The oxidation phenomenon may provide an important role in the sense. It could provide a “soft” oxide-like MoO_3_ which could serve to tether the nanoparticles to the magnesium alloy surface. However, if nano-MoS_2_ is overmuch oxidized into MoO_3_, the oxidation destroys the lubrication structure of layered MoS_2_ and thus results in the worse lubrication property [37]. Therefore, the synergistic lubricating effect enables the SiO_2_/MoS_2_ hybrid nanofluids to integrate the advantages and eradicate the disadvantages of SiO_2_ and MoS_2_ nanoparticles at optimal ratio. So the SiO_2_/MoS_2_ (0.25:0.75) hybrid nanoparticles exhibit superior lubricating performances than individual nano-SiO_2_ or nano-MoS_2_ even in high load and diverse velocity cases. Previous work on the combination of MoS_2_ with other nanoparticles as lubricant additives has reported improvements in their lubricating performance, such as nano-TiO_2_ [24], carbon nanotubes (CNT) [25], and graphene [26]. Since the testing method and analysis are completely different from the present study, comparable comparison between the results cannot be made. Even so, nano-SiO_2_ is less costly and facile preparation in contrast with the aforementioned nanoparticles. In conclusion, the SiO_2_/MoS_2_ hybrid nanoparticles are suggested as economic and environment friendly lubricant additives for the applications in forming process of magnesium alloy.

## Conclusions

In the present study, the tribological properties of the SiO_2_/MoS_2_ hybrid nanofluids to be used in metal forming fluids for magnesium alloy were investigated by a reciprocating sliding ball-on-plate contact configuration. Based on the experimental results, the following conclusions were drawn:

1. Compared with pure nanofluids, the SiO_2_/MoS_2_ hybrid nanofluids achieve lower friction coefficient and wear volume even in high load and diverse velocity cases for magnesium alloy/steel contacts under the test conditions.
2. The optimal SiO_2_/MoS_2_ mixing ratio and nanofluids concentration are 0.25:0.75 and 1.00–1.25 wt%, respectively. The 1.00 wt% SiO_2_/MoS_2_ (0.25:0.75) hybrid nanoparticles addition into the base oil shows reduction of friction coefficient by 46.6 % and reduction of wear volume by 50.5 % in contrast with the base oil.
3. The excellent lubrication properties of the SiO_2_/MoS_2_ hybrid nanoparticles are ascribed to the physical synergistic lubricating actions of nano-SiO_2_ and nano-MoS_2_ during the rubbing process. The synergistic lubricating effect enables the SiO_2_/MoS_2_ hybrid nanofluids to integrate the advantages and eliminate the disadvantages of SiO_2_ and MoS_2_ nanoparticles at optimal ratio.
